# The Application of Mitochondrial COI Gene-Based Molecular Identification of Forensically Important Scuttle Flies (Diptera: Phoridae) in Korea

**DOI:** 10.1155/2020/6235848

**Published:** 2020-09-28

**Authors:** Hajin Kim, Sang Eon Shin, Kwang Soo Ko, Seong Hwan Park

**Affiliations:** Department of Legal Medicine, Korea University College of Medicine, 02841 Seoul, Republic of Korea

## Abstract

Phoridae are a family of necrophagous flies commonly found in indoor death scene. They account for approximately 19.7% of the entomofauna in human cadavers in Korea. Additionally, this taxon is an indicator of indoor hygiene, and these flies appear in environments where access by other necrophagous insects is difficult, such as enclosed rooms. Thus, they are likely to be used as forensic evidence. Despite their importance in forensic investigations and environmental hygiene, detailed studies on the taxonomy and molecular barcoding for this family are scarce, including in Korea. Because accurate taxonomic information regarding necrophagous insects collected from a death-related scene is essential during medicolegal investigations, molecular barcoding data could be useful as well as reliable. In this paper, full-length nucleotide sequences of genes coding for the cytochrome c oxidase subunit I (COI) in 79 Phoridae larvae collected from 20 medicolegal autopsy cases in Korea were phylogenetically analyzed by comparing their sequences to the foreign barcoding data of Phoridae. Six mitochondrial haplogroups were identified, which two of them matched to foreign Phoridae fly species haplotypes, *Megaselia scalaris* (Loew, 1866) and *M. spiracularis* Schmitz 1938. Taxonomies of five other haplogroups, with nucleotide distances ranging from 1.68% to 2.26% from the *M. scalaris* group, could not be confirmed solely based on the molecular barcoding data. Further research should be performed to determine whether these five haplogroups are diverged conspecifics of *M. scalaris* or a closely related sister cryptic species of *M. scalaris*.

## 1. Introduction

**Figure 1:**
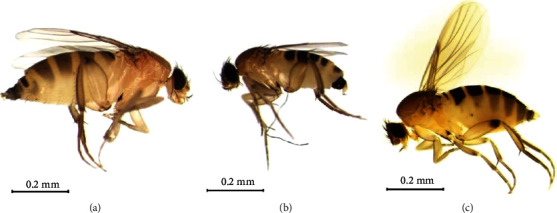
Lateral view of female *Megaselia scalaris* (a), male *M. scalaris* (b), and female *M. spiracularis* (c).

Necrophagous insect species are utilized as estimators of minimum postmortem intervals (mPMIs) in the medicolegal entomologic practice [1–4]. Various necrophagous dipteran species including those belonging to the families Calliphoridae and Sarcophagidae have been investigated based on their taxonomic characteristics and molecular barcoding [5–7]. Phoridae family or the scuttle fly family includes 230 genera and 4,000 species of flies that are often misidentified as fruit flies (family Drosophilidae) in human residences [8]. Flies of the Phoridae family have tiny black, brown, or yellowish bodies, humped backs, low small heads, and dark eyes. Costal veins extending only approximately halfway along the anterior wing margins are a characteristic feature. Additional characteristics were described by Disney [9], including the globose third antennal segment. Because they prefer indoor human waste as food, members of the family Phoridae could also be used as an important environmental hygiene indicator. Phorids are important indicators of mPMI, especially in indoor cases where access by large flies, such as those belonging to the family Calliphoridae or Sarcophagidae, is restricted. Despite their frequent occurrence, the systematics of phorids in Korea are still poorly understood. For instance, the large genus *Megaselia*, is not listed in the Korean catalog of insect fauna [10]. Phoridae were employed in approximately 19.7% of the autopsy cases (2015–2017 in Seoul, Incheon, and Gyeonggi provinces), which indicates that additional studies are required in this field [11]. Furthermore, little is known regarding the DNA barcoding data of this family. *Megaselia scalaris* (Loew, 1866) is the most well-known Phoridae species. Female *M. scalaris* can be identified by their sclerites in which segment six extends laterally on the abdomen [12]. Larvae of this species feed on an exceptionally broad range of decaying organic matter [13]. Male *Megaselia spiracularis* has an enlarged abdomen (Figure 1); the most prominent difference between *M. scalaris* and *M. spiracularis* is that a hairy mesopleuron is present in both male and female *M. spiracularis*, whereas it is bare in *M. scalaris* [14].

Because most samples recovered from medicolegal autopsies are in immature stages, the exact taxonomic information at the species level is rarely available unless the collected samples are reared to adulthood or have been molecularly barcoded. Because of the paucity of expert taxonomists and DNA barcoding data, taxonomic information regarding the collected Phoridae samples is rarely available. Moreover, it is not yet known how many species of the genus *Megaselia* infest human cadavers in Korea. In this study, we obtained full-length nucleotide sequences of genes coding for the cytochrome c oxidase subunit I (COI) in 79 Phoridae larvae collected from 20 medicolegal autopsies in Korea and compared them to previously published DNA barcoding results [15, 16] from the genus *Megaselia*.

## 2. Materials and Methods

### 2.1. Sample Collection

Experimental samples were obtained during autopsies or samples that had been stored from 2 to 4 years below -20°C in 70% ethanol that were used. A total of 79 samples were collected from 20 human cadavers in Seoul, Incheon, and Gyeonggi provinces in Korea. Seasonal changes had no effect on the presence of Phoridae, which occurred from February or April to December in 2015–2017. The samples were identified as Phoridae based on the morphological identification by observing the characteristic inferiorly directed posterior spiracles [17, 18]. The characteristics of Phoridae larvae were identified via stereomicroscopic observation. However, it was difficult to distinguish the stages and species of larvae merely by observing posterior spiracles because of the limitation associated with angular magnification and due to the minute sizes of the posterior spiracles.

### 2.2. DNA Extraction

Samples in 70% ethanol were dried on a paper towel for 5 min. After the larvae were chopped using cutter and forceps, DNA extraction was performed according to the manufacturer's instructions using an Exgene Tissue Mini Kit (GeneAll Biotechnology Co., Ltd., Seoul).

### 2.3. PCR and Sequencing

Primers were designed to amplify the entire length of the COI gene (Table 1). The reaction mixture for PCR contained AmpliTaq Gold DNA Polymerase (1 U; ThermoFisher Scientific, Inc., Foster City, CA, USA), 10× Gold Star buffer (2 *μ*l; premixed with MgCl_2_ and dNTPs), forward and reverse primers (4 pmoles), and template DNA (10 ng) in double distilled water, so that the final volume reached 20 *μ*l. The following PCR cycle was used: 95°C for 10 min (one cycle); 94°C for 1 min for denaturation, 51°C for 1 min for annealing, and 72°C for 1 min for extension (35 cycles); 60°C for 45 min for A-tailing (one cycle); and storage at 4°C until analyses. The optimum melting temperature (*T*_m_) of the primer sets was between 54°C and 60°C; however, as the experiment was initiated without knowing the exact species, the *T*_m_ was lowered until the primer sets could work universally well. After confirming the amplicon sizes by visualizing the bands on a 2% agarose gel, two-directional nucleotide sequencing—with the same primers that had been used for amplification—was conducted using a Big Dye Direct Sequencing Kit (ThermoFisher Scientific, Inc., Foster City, CA, USA).

Three primer pairs were used to amplify three different regions with overlaps to cover the complete COI gene. Primer pair F3-2/R3-2 was designed for samples for which amplification failure was observed using the primer pair F3/R3. The nucleotide sequence of the *Megaselia spiracularis* (MN832848) COI gene was used for designing the primers.

### 2.4. Phylogenetic Analysis and Sequence Comparison

Three contigs of the COI gene based on the amplicons generated by the three primer pairs were assembled to construct the full-length COI gene sequence using ChromasPro 2.1.5 software (Technelysium Pty Ltd., South Brisbane, Australia). Only COI gene regions were selected and aligned for further analysis. A neighbor-joining phylogenetic tree was constructed using MEGA X software [19]. To test the phylogeny, 1000 bootstrap replicates were used. Two Phoridae mitochondrial nucleotide sequences were employed for comparison (NC023794 M*. scalaris* and MN832848 M*. spiracularis*). Gene sequences indicative of *M. spiracularis* differed from MN832848 *M. spiracularis* by 0.4–0.5% and from NC023794 M*. scalaris* by 13.3–13.5% (Supplementary Table 1). The sequences indicative of *M. scalaris* differed from NC023794 *M. scalaris* by 1.2–2.6% and from MN832848 M*. spiracularis* by 13.6–14.7% (Supplementary Table 2). BLAST match scores of sequences indicative of *M. spiracularis* and *M. scalaris* to their conspecific reference sequences (MN832848 and NC023794) were 99.61-100% and 97.66-99.03%, respectively. *Lucilia sericata* (EU880208) COI was included as an outgroup. After defining the groups based on the clusters in the phylogenetic analysis, intragroup and intergroup mean nucleotide distances were calculated. Groups were defined as clusters that were more than 1% distant from one another. All obtained nucleotide sequences were submitted to GenBank (Table 2).

## 3. Results and Discussion

**Figure 2:**
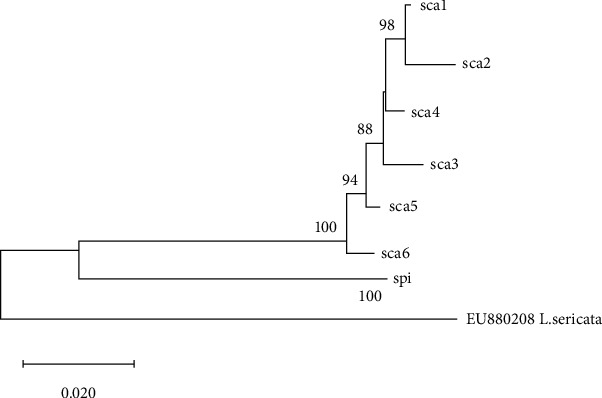
A schematic of a neighbor-joining phylogenetic tree using the sequences of the COI gene from 79 Phoridae larvae and two reference nucleotide sequences (NC023794 M*. scalaris* and MN832848 M*. spiracularis*) and an outgroup (EU880208 *Lucilia sericata*). NC023794 and MN832848 were clustered in sca5 and spi subgroups, respectively.

The schematic redrawing of a phylogenetic tree constructed using nucleotide sequences from 79 Korean Phoridae larvae revealed seven groups, i.e., sca1, sca2, sca3, sca4, sca5, sca6, and spi. Six groups—sca1 to sca6—clustered together away from the spi group with distances ranging from 11.64–12.38%. Previously published nucleotide sequences NC023794 (*M. scalaris*) and MN832848 (*M. spiracularis*) clustered in groups sca5 and spi, respectively (Figure 2). Mean intragroup distances ranged from 0.12–0.53%, whereas the intergroup distances ranged from 1.12–12.38% (Tables 3 and 4). The percent distances showed that the taxonomy for sca5 and spi groups follows that of *M. scalaris* and *M. spiracularis*, respectively. For sca1, sca2, sca3, sca4, and sca6 groups—as these groups exhibited a distance of 1.68–2.26% from sca5, an *M. scalaris* cluster—it was not possible to assume that these groups were conspecific with group sca5. Thus, at least two Phoridae species, *M. scalaris* and *M. spiracularis*, were identified in human cadavers in Korea. Because five groups were located in the gray zone with respect to species identification, it was impossible to classify these groups as variant haplogroups of *M. spiracularis* or heterologous sister species. The original phylogeny before the schematic redrawing is available as a supplementary figure (Supplementary Figure 1).

The number of base differences per site calculated by averaging over all sequence pairs between groups is shown. This analysis involved 79 nucleotide sequences. Codon positions included are 1^st^+2^nd^+3^rd^+noncoding. All ambiguous positions were removed for each sequence pair (pairwise deletion option). There were a total of 1539 positions in the final dataset.

There are no strict criteria that is determining the conspecificity or heterospecificity based on nucleotide sequence distances. Furthermore, nucleotide sequence distances between 1% and 2% were empirically considered as a gray zone, whereas when the distances >2%, the samples were generally accepted as being different species [20]. However, exceptionally high intraspecific distances have been reported in various taxa, including forensically important fly species. For example, DNA sequences of *Phormia regina* (Meigen, 1826) revealed exceptional intraspecific differences ranging from 3.52–4.31% between North American and West European samples [21]. Although we adopted the empirical criteria that distance >2% indicated heterospecificity, we could not be certain. Whether sca1, sca2, sca3, sca4, and sca6 groups clustered independently. They formed a large cluster with group sca5, which includes the previously reported sequence of the *M. scalaris* COI gene (NC023794). Therefore, it is not conclusive whether these five clusters could be attributed to five cryptic species or merely five conspecific variations because of large intraspecific distances. Cognato specified that the highest intraspecific distance among eight fly species was 3.5% based on the study of species *Phytomyza verticillatae* [22]. Because the sca1, sca2, sca3, sca4, sca5, and sca6 clusters only corresponded to *M. scalaris* samples collected from indoor cases, taxonomic studies on Phorid flies existing in indoor conditions in Korea are required. Furthermore, only two species, *M. scalaris* and *M. spiracularis*, showed significant BLAST scores with nucleotide sequences obtained in this study, and the species diversity of the genus *Megaselia* might be underestimated across the world, including Korea.

## 4. Conclusions

The nucleotide sequences of the COI genes from 79 necrophagous Phoridae flies recovered from human cadavers were analyzed. Based on this analysis, we confirmed the presence of COI gene sequences corresponding to those of two previously known species, *M. scalaris* and *M. spiracularis* and five haplogroups closely that are related to *M. scalaris* COI. Further taxonomic studies are required to reveal the necrophagous Phoridae fauna especially of genus *Megaselia* and to investigate the possible existence of cryptic species in Korea.

## Figures and Tables

**Table 1:** Primer information.

Primer name	Sequences	Binding locations
F1	5′-CCT TTA GAA TTG CAG TCT AAT GTC A-3′	tRNA-Cys
R1	5′-TAA ACT TCA GGG TGA CCA AAA AAT CA-3′	653-678 on COI
F2	5′-TTG TTA CTG CCC ATG CAT TT-3′	163-182 on COI
R2	5′-TGT TAA TCC CCC AAT TGT GAA-3′	1035-1055 on COI
F3	5′-AAA CCT TCG GTT CTC TTG GA-3′	740-759 on COI
R3	5′-AAT GGG GAA GCT CTA TCT TGA-3′	26-46 on COII
F3-2	5′-GAG CTC ATC ATA TAT TTA CTG TTG GAA-3′	812-838 on COI
R3-2	5′-ATT AGT GGA GAA GCT CTA TCT TGA AG-3′	24-49 on COII

**Table 2:** List of COI gene sequences for Phoridae flies obtained in this study.

Haplogroup name	Sample name	Sequence coverage on COI	Length (nucleotide)	Location	GenBank accession number	Reference
sca1	1-2, 1-3, 7-2, 10-3, 11-1, 12-1, 13-1, 13-2, 13-4, 14-1, 14-2, 14-3, 14-4, 15-4, 17-2, 17-3, 17-4, 19-2, 20-2	All	1,539 bp	South Korea	MT396274, MT396280, MT396287, MT296299, MT296304, MT396306, MT396320, MT396340-MT396343, MT396346, MT 396350-MT396351, MT396353-MT396354, MT396362-MT396364	Newly sequenced
sca2	3-3	All	1,539 bp	South Korea	MT396277	Newly sequenced
sca3	7-3, 8-1, 8-2, 8-4, 10-1, 10-4, 18-1, 18-2	All	1,539 bp	South Korea	MT396275-MT396276, MT396279, MT396290, MT396310, MT396313, MT396358-MT396359	Newly sequenced
sca4	1-1, 3-1, 3-2, 4-1, 4-2, 4-4, 5-3, 5-4, 11-2, 11-3, 11-4	All	1,539 bp	South Korea	MT396271-MT396272, MT396278, MT396282, MT396286, MT396291, MT396293, MT396295, MT396301, MT396307, MT396309	Newly sequenced
sca5	1-4, 2-1, 2-3, 2-4, 3-4, 4-3, 5-1, 7-4 8-3, 9-2, 10-2, 13-4, 15-1, 15-2, 15-3, 17-1, 19-1, 19-3, 19-4	All	1,539 bp	South Korea	MT396273, MT396292, MT396294, MT396298, MT396300, MT396302, MT396312, MT396314, MT396317-MT396319, MT396344-MT396345, MT396347, MT396349, MT396352, MT396361, MT396366-MT396367	Newly sequenced
sca6	2-2, 9-1, 9-3, 9-4, 12-2, 12-3, 12-4, 16-1, 16-2, 16-3, 16-4, 18-3, 18-4	All	1,539 bp	South Korea	MT396284-MT396285, MT396288, MT396303, MT396305, MT396308, MT396311, MT396348, MT396355-MT396357, MT396360, MT396365	Newly sequenced
Spi	5-2, 6-1, 6-2, 6-3, 6-4, 7-1, 20-1, 20-3	All	1,539 bp	South Korea	MT396281, MT396283, MT396289, MT396296, MT396297, MT 396315, MT396316, MT396321	Newly sequenced

**Table 3:** Percent distances of full-length COI gene sequences among seven Phoridae fly groups.

sca1	*—*						
sca2	*1.18*	*—*					
sca3	*1.59*	*2.22*	*—*				
sca4	*1.12*	*1.75*	*1.40*	*—*			
sca5	*1.69*	*2.26*	*2.10*	*1.68*	*—*		
sca6	*2.16*	*2.90*	*2.33*	*2.23*	*1.84*	*—*	
Spi	*12.09*	*12.38*	*11.98*	*11.83*	*11.87*	*11.64*	*—*
	sca1	sca2	sca3	sca4	sca5	sca6	Spi

**Table 4:** Intragroup percent distances of full-length COI gene sequences in seven Phoridae fly groups.

Group name	Intragroup differences
sca1	0.001709928
sca2	n/c^∗^
sca3	0.000997865
sca4	0.001228661
sca5	0.005312176
sca6	0.003115576
Spi	0.001137102

^∗^Intragroup distance is not available for sca2 because this cluster corresponded to a single sample.

## Data Availability

The data is full-length nucleotide sequences of the cytochrome c oxidase subunit I (COI) genes of necrophagous Phoridae in South Korea. These can be found at NCBI Genbank, and there is no restrictions on data access.

## Supplementary Materials

Supplementary Materials The original phylogeny before schematic redrawing is available as a supplementary figure: “See Supplementary Figure 1 in the Supplementary Material for comprehensive image analysis.” The detailed percent distances table between species and reference genes (NC023794 and MN832848) are available as Supplementary Tables 1 and 2.
