# Supplementary figures and images for: The Application of Mitochondrial COI Gene-Based Molecular Identification of Forensically Important Scuttle Flies (Diptera: Phoridae) in Korea
Source: Biomed Res Int. 2020 Sep 28;2020:6235848. doi: 10.1155/2020/6235848 (PMC7542508; doi:10.1155/2020/6235848)

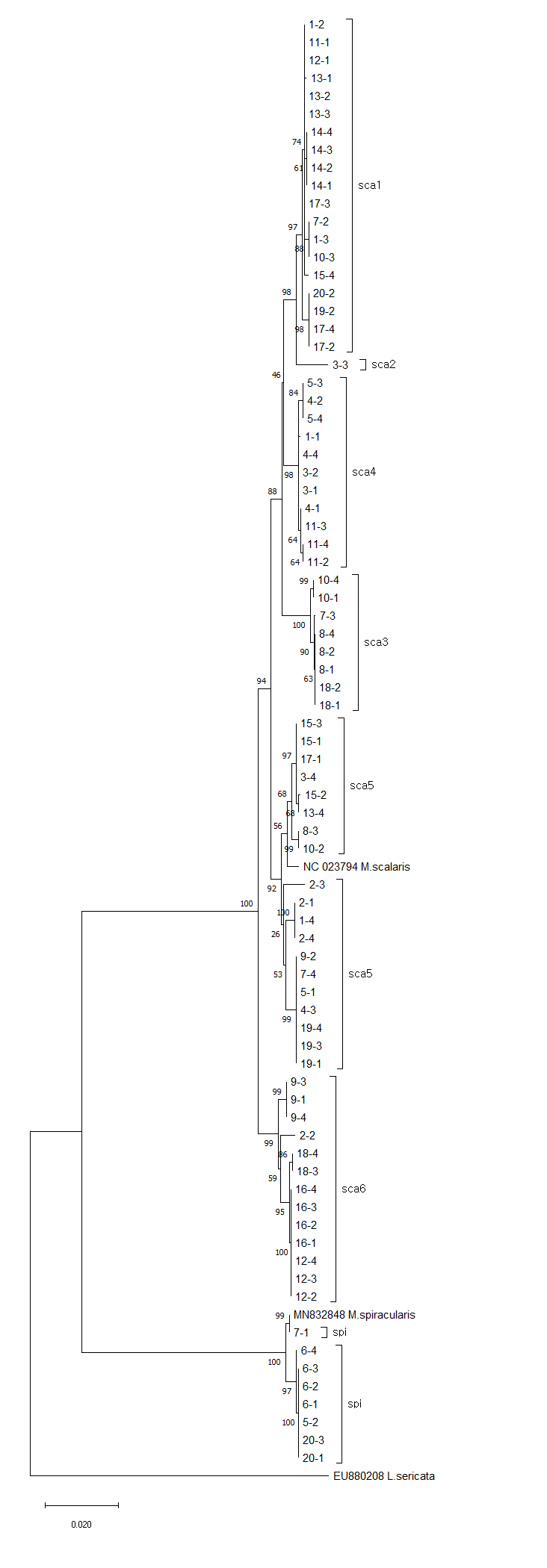

Supplement: Supplementary Materials — The original phylogeny before schematic redrawing is available as a supplementary figure: “See Supplementary Figure 1 in the Supplementary Material for comprehensive image analysis.” The detailed percent distances table between species and reference genes (NC023794 and MN832848) are available as Supplementary Tables 1 and 2. [file 6235848.f1.zip › 6235848 supplementary fig 1 - Phoridae NJ tree.png]
